# Development and Crossover Evaluation of an Artificial Intelligence‐Assisted System for Solid Pancreatic Lesion Detection and Pancreatic Parenchyma Recognition in Endoscopic Ultrasonography (With Video)

**DOI:** 10.1111/den.70282

**Published:** 2026-09-10

**Authors:** Toshio Fujisawa, Takamichi Kuwahara, Tatsuya Sato, Yusuke Takasaki, Nozomi Okuno, Ryunosuke Hakuta, Ko Tomishima, Shin Haba, Naminatsu Takahara, Yukiko Ito, Yousuke Nakai, Kazuo Hara, Hiroyuki Isayama

**Affiliations:** ^1^ Department of Gastroenterology, Graduate School of Medicine Juntendo University Tokyo Japan; ^2^ Department of Gastroenterology Aichi Cancer Center Nagoya Japan; ^3^ Department of Gastroenterology, Graduate School of Medicine The University of Tokyo Tokyo Japan; ^4^ Department of Internal Medicine, Institute of Gastroenterology Tokyo Women's Medical University Tokyo Japan; ^5^ Department of Gastroenterology Japanese Red Cross Medical Center Tokyo Japan

**Keywords:** artificial intelligence, endoscopic ultrasonography, novice, pancreatic parenchyma, solid pancreatic lesion

## Abstract

**Background and Study Aims:**

Pancreatobiliary endoscopic ultrasonography (EUS) is technically demanding, and supervised training opportunities are limited. We developed an artificial intelligence (AI) overlay system for detecting solid pancreatic lesions (SPL) and recognizing pancreatic parenchyma (PP) and evaluated its effect on reader performance.

**Patients and Methods:**

Across six centers, two deep learning‐based models were trained using expert‐annotated EUS frames. We then conducted a randomized, two‐sequence, two‐period crossover reader study in which eight endosonographers (five novices and three experts) interpreted image sets with and without AI assistance. The primary endpoint was superiority of sensitivity for SPL detection among novices; key secondary endpoints included specificity and PP recognition.

**Results:**

From 118 patients, 120 SPL‐positive/negative image sets and 160 PP‐positive/negative image sets were constructed. Among novices, AI assistance improved SPL detection sensitivity (88.7% vs. 76.8%, *p* < 0.001) and accuracy (86.4% vs. 78.7%), while specificity met the predefined noninferiority criterion (84.2% vs. 80.5%, *p* < 0.001). For PP recognition, sensitivity increased numerically (86.3% vs. 83.3%) but did not meet the predefined superiority criterion (*p* = 0.095); specificity met the noninferiority criterion (87.8% vs. 81.0%), and accuracy increased from 82.1% to 87.0%. Among experts, sensitivity was maintained for both tasks, whereas specificity increased with AI assistance.

**Conclusions:**

AI assistance improved SPL detection among novice endosonographers. For PP recognition, sensitivity increased without reaching statistical superiority, whereas specificity met the predefined noninferiority criterion. These findings support a potential adjunctive role for AI in EUS interpretation.

## Graphical abstract

Overview of the development and randomized crossover evaluation of an artificial intelligence (AI)‐assisted overlay system for endoscopic ultrasonography (EUS). The system was designed to support detection of solid pancreatic lesions (SPLs) and recognition of pancreatic parenchyma (PP). In a multicenter crossover reader study including novice and expert endosonographers, AI assistance improved SPL detection among novices and increased specificity while maintaining sensitivity among experts. For PP recognition, specificity improved, whereas the increase in sensitivity among novices did not reach statistical superiority. EUS, endoscopic ultrasonography; PP, pancreatic parenchyma; SPL, solid pancreatic lesion.

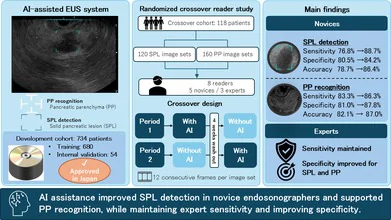

## 1 Introduction

Endoscopic ultrasonography (EUS) is essential for diagnosing pancreatic cancer and evaluating various pancreatobiliary diseases [1, 2]. However, pancreatobiliary EUS is technically challenging and is mainly performed by specialists at high‐volume centers [3, 4]. Considerable interindividual variation in pancreatic and biliary anatomy requires extensive training and experience to ensure accurate screening and diagnosis [5, 6, 7, 8]. Moreover, a shortage of experienced supervisors limits adequate training opportunities for novice endosonographers [9, 10].

Artificial intelligence (AI) has rapidly advanced in medicine and increasingly influences clinical practice [11]. In gastrointestinal endoscopy, AI‐based systems have been introduced to assist with polyp detection [12, 13, 14] and the differentiation of benign and malignant lesions [15, 16]. In the pancreatobiliary field, AI systems have also been developed for the detection and classification of pancreatic tumors [17, 18, 19], although their clinical implementation remains limited [20, 21].

We developed an AI‐assisted system for pancreatobiliary EUS with two functions: real‐time detection of solid pancreatic lesions (SPL) and recognition of pancreatic parenchyma (PP). The system, developed by FUJIFILM Corporation, has received regulatory approval in Japan as the EW10‐US01 Ultrasound Endoscopic Image Examination Support Program. Using a crossover reader study, we evaluated whether AI assistance improved the recognition performance of novice endosonographers.

## 2 Methods

### 2.1 Patients and EUS Procedures

Patients were prospectively enrolled at four centers in Japan and two centers in Europe. The AI development cohort was enrolled from April 2020 to September 2021, and the crossover trial cohort from October 2021 to November 2022. Eligible patients were aged ≥ 20 years, were scheduled to undergo EUS for evaluation of pancreatobiliary disease, and provided written informed consent. Patients considered unsuitable by the attending physician were excluded.

Solid pancreatic lesions (SPLs) included pancreatic ductal adenocarcinoma, neuroendocrine tumor, solid pseudopapillary neoplasm, and mass‐forming chronic pancreatitis. Neoplastic lesions were confirmed pathologically by EUS‐guided tissue acquisition and/or surgery. Mass‐forming chronic pancreatitis was diagnosed by the absence of malignancy on tissue sampling and/or lesion stability for at least 6 months on imaging [22, 23].

EUS was performed under conscious sedation using an SU‐1 ultrasound system with an EG‐580UT or EG‐740UT curved linear‐array echoendoscope (FUJIFILM Corporation, Tokyo, Japan), and examination videos were recorded.

The study was approved by the Institutional Review Board of Juntendo University (No. H19‐0225) and conducted in accordance with the Declaration of Helsinki and Japanese Good Clinical Practice requirements. The study design was discussed with the Pharmaceuticals and Medical Devices Agency before study initiation.

### 2.2 AI Development and Internal Validation

Patients enrolled from April 2020 to September 2021 were assigned to training and internal validation cohorts. Expert endoscopists annotated pancreatic parenchyma (PP) and SPLs on each frame using polygonal contours, which were converted into rectangular bounding boxes for model training.

Two one‐stage convolutional neural network‐based object‐detection models were developed: one for SPL detection and one for PP recognition [24, 25]. Each model generated a rectangular bounding box around the predicted target region. The internal validation cohort was used for model development and standalone AI evaluation using separate SPL and PP datasets.

### 2.3 Crossover Reader Trial

For the crossover cohort, medical image engineers at an independent contract research organization (CRO) reviewed full‐length EUS videos and extracted sets of 12 consecutive frames from SPL‐positive, SPL‐negative, PP‐positive, and PP‐negative scenes.

Three senior pancreatobiliary endoscopists who did not participate in the reader trial independently annotated SPL and PP in each frame. The consensus reference region of interest was defined as the mean coordinates of the three bounding boxes. For target‐negative frames, all three experts confirmed target absence. Spatial agreement was evaluated using mean intersection over union (IoU), calculated separately for SPL and PP.

**FIGURE 1:**
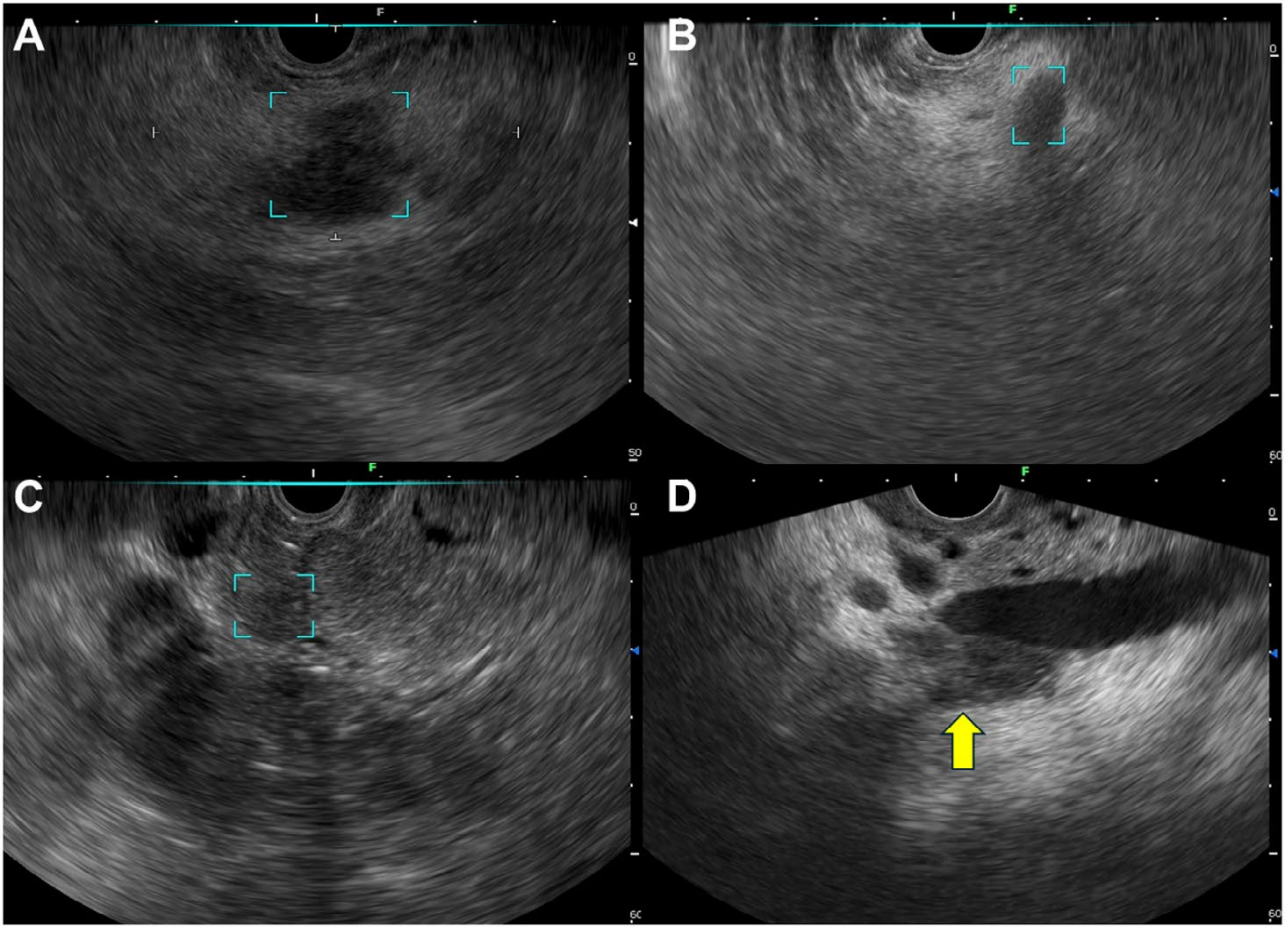
Representative AI‐assisted EUS images and examples of misdetection. (A) Simultaneous AI recognition of the pancreatic parenchyma and detection of a solid pancreatic lesion. White markers indicate the pancreatic parenchyma, and blue corner markers indicate the suspected lesion. (B) False‐positive detection of an incompletely anechoic vascular structure. (C) False‐positive detection caused by heterogeneous pancreatic parenchyma. (D) False‐negative detection of a solid pancreatic lesion, indicated by the yellow arrow.

The AI models were applied to the four datasets to generate assisted and unassisted images (Figure 1A and Video S1). Eight readers participated in a randomized, two‐sequence, two‐period, multi‐reader, multi‐case crossover trial: three experts and five novices. Experts were Japan Pancreas Society‐certified instructors or physicians with equivalent experience. Novices had performed > 100 supervised EUS procedures but < 250 independent procedures. The upper threshold was based on published competency benchmarks indicating that approximately 225–250 procedures are required before competency assessment. Reader experience is shown in Table S1.

During the first period, readers evaluated half of the image sets with AI assistance and half without assistance. After a 4‐week washout period, selected based on previous AI crossover reader studies, the same sets were evaluated under the opposite condition. Randomization was generated using validated software, and allocation was concealed by the CRO until database lock. Readers were blinded to the reference annotations and recorded target presence or absence, drawing a bounding box when present.

After study design finalization, image processing, data management, randomization, statistical analysis, and preparation of the clinical study report were performed by the independent CRO.

### 2.4 Outcome Definitions

Detection performance was evaluated using IoU. At the frame level, a detection was considered a true positive when IoU was ≥ 0.30 and a false negative when IoU was < 0.30 or no detection was generated. A detection in a target‐negative frame was considered a false positive, whereas the absence of both target and detection was considered a true negative.

The prespecified IoU threshold of 0.30 was selected because the system was designed to provide approximate visual alerts rather than precise segmentation. This threshold is consistent with expert consensus for endoscopic computer‐aided detection and was considered clinically appropriate by participating EUS experts.

At the image‐set level, a positive set was correctly detected when at least two consecutive frames had an IoU ≥ 0.30. Sensitivity, specificity, accuracy, positive predictive value, and negative predictive value were calculated accordingly.

The primary endpoint was superiority of set‐level sensitivity for SPL detection by novices with versus without AI assistance. Secondary endpoints included noninferiority of SPL specificity among novices with a margin of −8 percentage points; superiority of PP sensitivity and noninferiority of PP specificity among novices, with a specificity margin of −10 percentage points; other diagnostic performance measures among novices and experts; and standalone AI performance.

### 2.5 Sample Size Estimation

Based on pilot data comparing SPL detection sensitivity between novices without AI and standalone AI, at least 55 image sets were required with a two‐sided α of 0.05 and 80% power. To improve robustness, at least 120 sets were planned.

### 2.6 Statistical Analysis

Analyses were performed using R version 4.3.1 and SAS version 9.4. Superiority was evaluated using two‐sided tests with *p* < 0.05, whereas noninferiority was evaluated using one‐sided tests with prespecified margins. Fisher's exact test and the Mann–Whitney *U* test were used for categorical and continuous variables, respectively.

For novice SPL detection, superiority of sensitivity was assessed using the chi‐squared test, and noninferiority of specificity was assessed using the Farrington–Manning test with a margin of −8 percentage points. The same methods were used for PP recognition, with a −10‐percentage‐point margin for specificity. Sensitivity, specificity, and accuracy are reported with 95% confidence intervals. An exploratory post hoc power analysis was conducted for PP sensitivity among novices using the observed difference and a two‐sided significance level of 0.05.

## 3 Results

### 3.1 Patient Flow and Extraction of EUS Images

As shown in Figure S1, 734 patients were assigned to the development cohort, comprising 680 patients in the training cohort and 54 in the validation cohort. The training cohort yielded 91,550 images for SPL detection and 202,400 images for PP recognition. The validation cohort comprised 29 patients with SPL and 25 without SPL, yielding 694 images for SPL evaluation (237 SPL positive and 457 SPL negative) and 316 images for PP recognition (163 PP positive and 153 PP negative).

**FIGURE 2:**
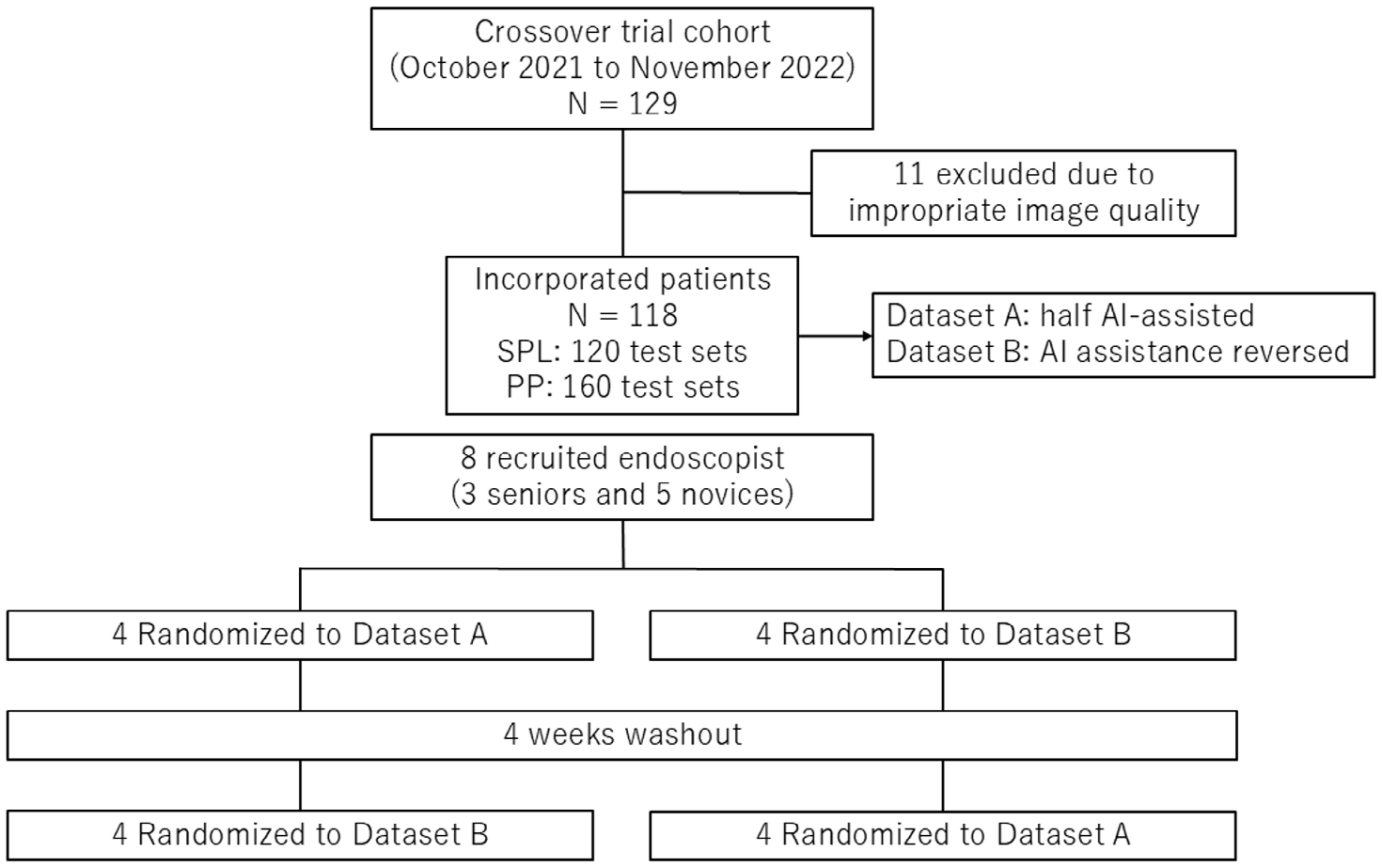
Patient flow in the crossover trial. Of 129 patients recruited, 11 were excluded due to inadequate image quality, leaving 118 patients who provided 120 solid pancreatic lesions sets and 160 pancreatic parenchyma sets. From the entire set of 280 images (including both SPL and PP), Dataset A was created with half of the images AI‐assisted, and Dataset B was generated by reversing the AI assistance. Eight endoscopists (three experts, five novices) participated in a two‐period crossover trial: Four were initially allocated to Dataset A and the other four to Dataset B, then crossed over after a 4‐week washout.

Of 129 patients in the crossover trial cohort, 11 were excluded because of inadequate image quality (Figure 2). The remaining 118 patients provided 120 SPL‐positive/negative image sets and 160 PP‐positive/negative image sets, each consisting of 12 consecutive frames.

### 3.2 Background Characteristics of the Patients and Images

The background characteristics of the training and validation cohorts are presented in Tables S2 and S3, respectively.

The characteristics of the crossover trial image sets are shown in Table 1. The cohort comprised 118 patients, including 58 women (49.2%), and the most frequently represented age group was the 70s. The 120 SPL image sets included 89 pancreatic ductal adenocarcinomas (74%), 16 neuroendocrine tumors, 13 cases of mass‐forming chronic pancreatitis, and two solid pseudopapillary neoplasms. The most common tumor size was 25–30 mm, and the pancreatic body was the most frequent tumor location.

**TABLE 1:** Background characteristics of test question image sets in the crossover trial.

Detection of soild pancraetic lesions (*n* = 120)		Recognition of pancreatic parenchyma (*n* = 160)	
**Diagnosis of the solid lesions**		**Conditions of the pancreatic parenchyma**	
Pancreatic ductal adenocarcinoma	89 (74%)	Normal	109 (68%)
Neuroendocrine neoplasm	16 (13%)	Chronic pancreatitis	22 (14%)
Mass‐forming pancreatitis	13 (11%)	Fatty pancreas	14 (9%)
Solid pseudopapillary neoplasm	2 (2%)	Unknown	15 (9%)
**Size of the lesions**		**Approach sites**	
< 15 mm	18 (15%)	Stomach	68 (43%)
15 ≤ < 20 mm	13 (11%)	Bulbs of duodenum	58 (36%)
20 ≤ < 25 mm	24 (20%)	Descending of duodenum	34 (21%)
25 ≤ < 30 mm	26 (22%)		
30 ≤ < 35 mm	20 (17%)		
35 ≤ < 40 mm	11 (9%)		
40 mm ≤	8 (7%)		
**Location of the lesions**			
Head	41 (34%)		
Body	43 (36%)		
Tail	32 (27%)		
Others	4 (3%)		

Among the 160 PP image sets, 109 (68%) showed normal parenchyma, whereas chronic pancreatitis and fatty pancreas accounted for 14% and 9%, respectively. The transgastric approach was most common, and the pancreatic head was the most frequently observed region (47%).

The detailed composition of the 120 SPL‐negative image sets is shown in Table S4. Of these, 90 sets (75.0%) represented nonlesion pancreatic areas in patients with SPL. The background pancreatic findings were normal in 82 sets (68.3%), fatty pancreas in 15 (12.5%), pancreatitis in 17 (14.2%), and unknown in 6 (5.0%).

### 3.3 Interrater Agreement Among Senior Expert Annotators

Interrater agreement among the three senior expert endoscopists was evaluated using the mean intersection over union (IoU) of independently drawn bounding boxes. After excluding cases with IoU < 0.30, the mean IoU was 0.73 for SPL and 0.69 for PP, indicating reasonable spatial agreement among the annotators.

### 3.4 Effect of AI Assistance on SPL Detection in the Crossover Trial

Without AI assistance, novices detected SPLs with a sensitivity of 76.8%, specificity of 80.5%, and accuracy of 78.7% (Table 2). With AI assistance, sensitivity increased to 88.7% (*p* < 0.001 for superiority), specificity to 84.2% (*p* < 0.001 for noninferiority), and accuracy to 86.4%. Thus, the primary endpoint of sensitivity superiority and the secondary endpoint of specificity noninferiority were achieved.

**TABLE 2:** Detection of solid pancreatic lesions in the crossover trial.

Participants	Sensitivity	Specificity	PPV	NPV	Accuracy
	[95% CI]	[95% CI]	[95% CI]	[95% CI]	[95% CI]
AI only	92.5%	80.8%	82.8%	91.5%	86.7%
	[87.8–97.2]	[73.8–87.9]	[76.5–89.2]	[86.2–96.8]	[82.4–91.0]
Novice without assistance	76.8%	80.5%	79.8%	77.7%	78.7%
	[73.5–80.2]	[77.3–83.7]	[76.5–83.0]	[74.4–80.9]	[76.3–81.0]
Novice with AI assistance	88.7%	84.2%	84.8%	88.1%	86.4%
	[86.1–91.2]	[81.2–87.1]	[82.0–87.7]	[85.5–90.8]	[84.5–88.4]
*p* value	< 0.001	< 0.001	0.020	< 0.001	< 0.001
Expert without assistance	93.6%	55.6%	67.8%	89.7%	74.6%
	[91.1–96.1]	[50.4–60.7]	[63.7–71.9]	[85.7–93.7]	[71.4–77.8]
Expert with AI assistance	92.2%	66.9%	73.6%	89.6%	79.6%
	[89.5–95.0]	[62.1–71.8]	[69.5–77.7]	[85.9–93.2]	[76.6–82.5]
*p* value	0.470	0.001	0.050	0.970	0.024

Abbreviations: AI, artificial intelligence; CI, confidence interval; NPV, negative predictive value; PPV, positive predictive value.

Among experts, sensitivity, specificity, and accuracy were 93.6%, 55.6%, and 74.6%, respectively, without AI assistance and 92.2%, 66.9%, and 79.6%, respectively, with AI assistance. Standalone AI achieved a sensitivity of 92.5%, specificity of 80.8%, positive predictive value (PPV) of 82.8%, negative predictive value (NPV) of 91.5%, and accuracy of 86.7%.

At the individual‐reader level, all five novices showed improved sensitivity and accuracy with AI assistance (Table S5). Among experts, sensitivity remained high, while specificity improved in all three readers, resulting in higher accuracy.

### 3.5 Effect of AI Assistance on PP Recognition in the Crossover Trial

Without AI assistance, novices recognized PP with a sensitivity of 83.3%, specificity of 81.0%, and accuracy of 82.1% (Table 3). With AI assistance, sensitivity increased numerically to 86.3%, but the predefined criterion for superiority was not met (*p* = 0.095). Specificity increased to 87.8% and met the predefined criterion for noninferiority (*p* < 0.001), while accuracy increased to 87.0%. An exploratory post hoc power analysis for the PP sensitivity endpoint yielded a power of 0.386.

**TABLE 3:** Recognition of pancreatic parenchyma in the crossover trial.

Participants	Sensitivity	Specificity	PPV	NPV	Accuracy
	[95% CI]	[95% CI]	[95% CI]	[95% CI]	[95% CI]
AI only	90.6%	86.3%	86.8%	90.2%	88.4%
	[86.1–95.1]	[80.9–91.6]	[81.7–92.0]	[85.5–94.9]	[84.9–91.9]
Novice without assistance	83.3%	81.0%	81.4%	82.9%	82.1%
	[80.7–85.8]	[78.3–83.7]	[78.8–84.1]	[80.2–85.5]	[80.2–84.0]
Novice with AI assistance	86.3%	87.8%	87.6%	86.5%	87.0%
	[83.9–88.6]	[85.5–90.0]	[85.3–89.9]	[84.1–88.8]	[85.4–88.6]
*p* value	0.095	< 0.001	< 0.001	0.047	< 0.001
Expert without assistance	87.3%	81.0%	82.2%	86.4%	84.2%
	[84.3–90.3]	[77.5–84.5]	[78.8–85.5]	[83.3–89.6]	[81.9–86.5]
Expert with AI assistance	88.5%	87.5%	87.6%	88.4%	88.0%
	[85.7–91.4]	[84.5–90.5]	[84.7–90.6]	[85.5–91.3]	[86.0–90.1]
*p*	0.550	0.006	0.016	0.364	0.015

Abbreviations: AI, artificial intelligence; CI, confidence interval; NPV, negative predictive value; PPV, positive predictive value.

Among experts, sensitivity, specificity, and accuracy were 87.3%, 81.0%, and 84.2%, respectively, without AI assistance and 88.5%, 87.5%, and 88.0%, respectively, with AI assistance. Standalone AI achieved a sensitivity of 90.6%, specificity of 86.3%, PPV of 86.8%, NPV of 90.2%, and accuracy of 88.4%.

At the individual‐reader level, most novices showed improved sensitivity, specificity, and accuracy with AI assistance, although sensitivity decreased in one reader and specificity decreased in another (Table S6). Among experts, specificity and accuracy were maintained or improved in all three readers, whereas one expert showed a slight decrease in sensitivity.

### 3.6 Performance of Standalone AI in the Internal Validation

In the internal validation datasets, the mean IoU of true‐positive detections was 0.775 for SPL and 0.734 for PP. At the prespecified frame‐level IoU threshold of ≥ 0.30, standalone AI achieved a sensitivity of 93.7% (95% confidence interval [CI], 90.6%–96.8%), specificity of 97.2% (95% CI, 95.6%–98.7%), and accuracy of 96.0% (95% CI, 94.5%–97.4%) for SPL detection (Table 4).

**TABLE 4:** Results of AI performance in the validation cohort.

AI only	Sensitivity	Specificity	PPV	NPV	Accuracy
	[95% CI]	[95% CI]	[95% CI]	[95% CI]	[95% CI]
Detection of solid pancreatic lesions	93.7%	97.2%	94.5%	96.7%	96.0%
	[90.6–96.8]	[95.6–98.7]	[91.5–97.4]	[95.1–98.4]	[94.5–97.4]
Recognition of pancreatic parenchyma	95.1%	96.7%	96.9%	94.9%	95.9%
	[91.8–98.4]	[93.9–99.5]	[94.2–99.6]	[91.4–98.3]	[93.7–98.1]

Abbreviations: AI, artificial intelligence; CI, confidence interval; NPV, negative predictive value; PPV, positive predictive value.

For PP recognition, sensitivity was 95.1% (95% CI, 91.8%–98.4%), specificity was 96.7% (95% CI, 93.9%–99.5%), and accuracy was 95.9% (95% CI, 93.7%–98.1%). Representative false‐positive and false‐negative AI detections are shown in Figure 1B–D.

## 4 Discussion

In this crossover trial, AI assistance improved SPL detection among novices, increasing sensitivity by approximately 12 percentage points and accuracy by 8 percentage points, while meeting the predefined noninferiority criterion for specificity. For PP recognition, sensitivity increased modestly but did not meet the predefined criterion for superiority, whereas specificity met the noninferiority criterion and accuracy improved. Among experts, sensitivity was maintained for both tasks, while specificity increased, suggesting that AI assistance may reduce over‐detection without compromising sensitivity.

Standalone AI showed slightly higher sensitivity than AI‐assisted novices for SPL detection, whereas AI‐assisted novices showed slightly higher specificity. This difference may reflect the complementary roles of AI and endoscopists. The AI system sensitively presents candidate lesions, while the endoscopist integrates the alerts with morphological features on EUS images and makes the final decision. Thus, the system may be most appropriately used as a second observer rather than as an autonomous diagnostic tool. However, because this comparison was not prespecified, this interpretation remains exploratory.

The relatively low SPL specificity among experts without AI assistance may reflect a sensitivity‐prioritized reading strategy intended to avoid missed lesions. In clinical practice, experts may initially regard subtle findings as suspicious and subsequently reassess them from different angles before reaching a final diagnosis. AI assistance may have helped reduce this initial over‐detection. Nevertheless, the effects varied among individual readers, with reduced sensitivity observed in one novice and one expert, indicating that the benefit of AI also depends on how its output is interpreted and incorporated into decision‐making.

Exploratory review of misdetections suggested that heterogeneous pancreatic parenchyma and incompletely anechoic vascular or cystic structures may contribute to false‐positive SPL alerts. No consistent features associated with false‐negative detections were identified because few such examples were available. Representative cases are shown in Figure 1B–D, although systematic characterization of AI failure modes will require larger datasets.

Previous studies have demonstrated the potential of AI for SPL detection and PP recognition on EUS. Tonozuka et al. reported an area under the curve of 0.94 for pancreatic tumor detection using a model trained on 93 patients [19]. Zhang et al. developed a UNet++ model for PP segmentation using 294 EUS images, achieving a sensitivity of 0.984 and precision of 0.824 in external validation [26]. Robles et al. developed YOLOv4‐based models for recognizing normal EUS anatomy, with mean average precision values of 0.887 for linear EUS and 0.835 for radial EUS [27]. However, these studies generally used relatively small datasets and primarily evaluated standalone AI performance in offline settings [26, 27, 28, 29].

In contrast, the present study used a randomized crossover, multi‐reader, multi‐case design to directly quantify the incremental effect of AI assistance on physician performance. Inclusion of both novices and experts allowed us to assess how the effect differed according to reader experience. In addition, the development and validation datasets were obtained from multiple institutions in Japan and Europe, supporting the generalizability of the system. The system has also received regulatory approval and has been commercialized in Japan, distinguishing it from many previously reported models that remain at the developmental stage. Nevertheless, regulatory approval and commercialization should not be interpreted as evidence of clinical effectiveness during live EUS examinations.

An important distinction exists between the standardized offline evaluation used in this study and real‐time clinical EUS. The reader study used short image sets comprising 12 consecutive frames, whereas actual EUS examinations require continuous image interpretation, echoendoscope manipulation, and integration of clinical history, laboratory findings, and previous imaging. Therefore, the present results demonstrate performance under controlled reader‐study conditions but do not establish real‐time clinical effectiveness. Prospective studies during live EUS procedures are required.

This study has several limitations. First, only five novices and three experts participated, which may limit generalizability. Patient height and weight were not collected; therefore, the potential influence of body mass index on PP recognition could not be assessed. Second, although a 4‐week washout period was used to reduce recall and carryover, formal period and sequence‐by‐treatment interaction effects could not be reliably evaluated because of the limited number of readers and the available data structure. Residual period or carryover effects therefore cannot be completely excluded. Third, the consensus reference standard was based on annotations by three senior experts and may have introduced reference‐standard bias despite reasonable spatial agreement. Fourth, misdetection features were not systematically or quantitatively analyzed, and interpretability methods such as class activation maps or attention heatmaps were not available; therefore, the image features underlying AI predictions remain incompletely understood. Finally, improved recognition performance was not linked to downstream diagnostic accuracy, treatment decisions, or patient outcomes.

In conclusion, this randomized crossover reader study demonstrated that AI assistance improved SPL detection among novice endosonographers. For PP recognition, sensitivity increased numerically without meeting the predefined superiority criterion, whereas specificity met the predefined noninferiority criterion. These findings support a potential adjunctive role for AI in EUS interpretation, although its effectiveness during real‐time clinical examinations requires further investigation.

## Author Contributions

K.H., Y.N., and H.I.: conception of the work. T.F. and T.K.: design of the work and acquisition of data for the work. Y.I. and N.T.: analysis of data for the work. T.S., Y.T., N.O., R.H., K.T., S.H.: interpretation of data and drafting for the work. Y.I., N.T., K.H., Y.N., and H.I.: critical revision of the work for important intellectual content. All authors have participated sufficiently in the work to agree to be accountable for all aspects of the work in ensuring that questions related to the accuracy or integrity of any part of the work are appropriately investigated and resolved. All authors give final approval of the work to be submitted and published.

## Funding

FUJIFILM Corporation provided financial support for this study and participated in discussions regarding the study design before its finalization. After the study design had been finalized, the sponsor had no involvement in image‐data processing, test‐data management, allocation or randomization, statistical analysis, interpretation of the study results, preparation or revision of the manuscript, or the decision to submit the manuscript for publication.

## Ethics Statement

Approval of the research protocol by an Institutional Reviewer Board: This study was approved by the Institutional Review Board of Juntendo University (No. H19‐0225).

Registry and the registration no. of the study/trial: This study was conducted as a medical device clinical trial in accordance with the Japanese Good Clinical Practice requirements, with the study design discussed between FUJIFILM Corporation and the Pharmaceuticals and Medical Devices Agency before study initiation.

## Consent

All participating patients provided written informed consent.

## Conflicts of Interest

N.T., Y.N., K.H., and H.I. received a research grant from FUJIFILM Corporation, and Y.N. received an honorarium from FUJIFILM Corporation.

## Supporting information


**Figure S1:** Patient flow chart for AI engine development. Of 734 recruited patients, 680 were assigned to the training cohort (yielding 91,550 solid pancreatic lesions and 202,400 pancreatic parenchyma images for AI training) and 54 to the validation cohort (yielding 694 solid pancreatic lesions and 316 pancreatic parenchyma images for performance validation).

**Table S1:** EUS observation experience of each participant.

**Table S2:** Background characteristics of patients in the training cohort.

**Table S3:** Background characteristics of the images in the validation cohort.

**Table S4:** Composition of the SPL‐negative image sets in the crossover trial.

**Table S5:** Individual results of participants in the detection of solid pancreatic lesions.

**Table S6:** Individual results of participants in the recognition of pancreatic parenchyma.

**Video S1:** Representative video of AI‐assisted EUS. Blue corner markers indicate SPLs with minimal delay, and white markers highlight the pancreatic parenchyma almost simultaneously with its appearance.

## Data Availability

The data that support the findings of this study are available from the corresponding author upon reasonable request.
